# Diagnostic Performance of PET or PET/CT with Different Radiotracers in Patients with Suspicious Lung Cancer or Pleural Tumours according to Published Meta-Analyses

**DOI:** 10.1155/2020/5282698

**Published:** 2020-04-25

**Authors:** Filippo Lococo, Barbara Muoio, Marco Chiappetta, Dania Nachira, Leonardo Petracca Ciavarella, Stefano Margaritora, Giorgio Treglia

**Affiliations:** ^1^Università Cattolica del Sacro Cuore, Rome, Italy; ^2^Thoracic Surgery, Fondazione Policlinico Universitario A. Gemelli IRCCS, Rome, Italy; ^3^Clinic of Oncology, Oncology Institute of Southern Switzerland, Ente Ospedaliero Cantonale, Bellinzona, Switzerland; ^4^Clinic of Nuclear Medicine and Molecular Imaging, Imaging Institute of Southern Switzerland, Ente Ospedaliero Cantonale, Belllinzona and Lugano, Switzerland; ^5^Department of Nuclear Medicine and Molecular Imaging, Lausanne University Hospital and University of Lausanne, Lausanne, Switzerland; ^6^Health Technology Assessment Unit, Academic Education, Research and Innovation Area, Ente Ospedaliero Cantonale, Bellinzona, Switzerland

## Abstract

**Purpose:**

Several meta-analyses have reported data about the diagnostic performance of positron emission tomography or positron emission tomography/computed tomography (PET or PET/CT) with different radiotracers in patients with suspicious lung cancer (LC) or pleural tumours (PT). This review article aims at providing an overview on the recent evidence-based data in this setting.

**Methods:**

A comprehensive literature search of meta-analyses published in PubMed/MEDLINE and Cochrane Library database from January 2010 through March 2020 about the diagnostic performance of PET or PET/CT with different radiotracers in patients with suspicious LC or PT was performed. This combination of keywords was used: (A) “PET” OR “positron emission tomography” AND (B) “lung” OR “pulmonary” OR “pleur^∗^” AND (C) meta-analysis. Only meta-analyses on PET or PET/CT in patients with suspicious LC or PT were selected.

**Results:**

We have summarized the diagnostic performance of PET or PET/CT with fluorine-18 fluorodeoxyglucose (^18^F-FDG) and other radiotracers taking into account 17 meta-analyses. Evidence-based data demonstrated a good diagnostic performance of ^18^F-FDG PET or PET/CT for the characterization of solitary pulmonary nodules (SPNs) or pleural lesions with overall higher sensitivity than specificity. Evidence-based data do not support the routine use of dual time point (DTP) ^18^F-FDG PET/CT or fluorine-18 fluorothymidine (^18^F-FLT) PET/CT in the differential diagnosis of SPNs. Even if ^18^F-FDG PET/CT has high sensitivity and specificity as a selective screening modality for LC, its role in this setting remains unknown.

**Conclusions:**

Evidence-based data about the diagnostic performance of PET/CT with different radiotracers for suspicious LC or PT are increasing, with good diagnostic performance of ^18^F-FDG PET/CT. More prospective multicenter studies and cost-effectiveness analyses are warranted.

## 1. Introduction

Positron emission tomography (PET) is a noninvasive functional imaging method currently used for several oncological indications. This method can early detect pathophysiological changes in affected tissues in oncological patients, including patients with lung cancer (LC) or pleural tumours (PT), and these functional changes may occur before anatomical changes detected by conventional radiological techniques. Currently, hybrid imaging techniques as positron emission tomography/computed tomography (PET/CT) may provide combined functional and morphological information for early diagnosis of LC or PT [1].

Fluorine-18 fluorodeoxyglucose (^18^F-FDG) is the most used PET radiotracer in oncology: this radiolabelled glucose analogue is taken up by the cells via cell membrane glucose transporters and subsequently phosphorylated by hexokinase inside the cells. The ability of ^18^F-FDG PET or PET/CT to identify tumour lesions is mainly related to the increased glycolytic activity of the majority of tumour cells [1]. Beyond ^18^F-FDG, other PET radiotracers evaluating different metabolic pathway or receptor status have been used and approved for oncological indications [2].

Meta-analysis is a statistical technique for combining the findings of independent studies included in a systematic review and it is often used to assess the diagnostic performance of imaging methods. All meta-analyses are actually systematic reviews with components of statistical pooling of data [3]. Several meta-analyses have been published about the diagnostic performance of PET or PET/CT with different radiotracers in oncology [3].

The aim of this review article is to provide an overview of the findings of recent meta-analyses about the diagnostic performance of PET or PET/CT with different radiotracers in patients with suspicious LC or PT.

## 2. Methods

A comprehensive literature search of PubMed/MEDLINE and Cochrane Library databases was performed to find recently published meta-analyses on the diagnostic performance of PET or PET/CT with different radiotracers in patients with suspicious LC or PT.

A search algorithm based on the combination of the following terms was used: (A) “PET” OR “positron emission tomography” AND (B) “lung” OR “pulmonary” OR “pleur^∗^” AND (C) meta-analysis. The literature search was updated until March 25, 2020. No language restriction was used. Recent meta-analyses (published from 2010 until the last search date) investigating the diagnostic performance of PET or PET/CT by using different radiotracers in patients with suspicious LC or PT were eligible for inclusion. Titles and abstracts of the retrieved meta-analyses were reviewed, applying the inclusion criteria mentioned above.

For each selected meta-analysis, information was collected about the indication of PET or PET/CT (including the used radiotracer), authors, year of publication, number of original articles included, number of patients or lesions included, and pooled diagnostic performance measures with 95% confidence interval values (95% CI) if available. Finally, the main findings of the selected meta-analyses were briefly described.

## 3. Results

From the comprehensive computer literature search from PubMed/MEDLINE and Cochrane Library databases, 17 meta-analyses were selected and retrieved in full-text version [4–20]. The characteristics of the selected articles are summarized and presented in Table 1 and summarized as follows.

In particular, we have summarized: (1) the diagnostic performance of ^18^F-FDG PET or PET/CT as a screening method for LC; (2) the diagnostic performance of single time point (STP) and dual time point (DTP) ^18^F-FDG PET or PET/CT for characterization of solitary pulmonary nodules (SPNs); (3) the diagnostic performance of fluorine-18 fluorothymidine (^18^F-FLT) PET or PET/CT for characterization of SPNs; and (4) the diagnostic performance of ^18^F-FDG PET or PET/CT for characterization of pleural lesions.

### 3.1. Lung Cancer Screening

Chien et al. [4] conducted a meta-analysis to describe the role of ^18^F-FDG PET in LC screening. Four studies reported evidence of LC screening programs with selective ^18^F-FDG PET with a pooled sensitivity and specificity of 83% and 91%, respectively. Even if ^18^F-FDG PET has high sensitivity and specificity as a selective screening modality, the role of primary ^18^F-FDG PET or PET/CT screening for LC remains unknown. Further studies must be conducted to evaluate the use of this imaging method as screening modality for high-risk populations, preferably using randomized trials or prospective registration.

### 3.2. Characterization of Solitary Pulmonary Nodules with Single Time Point ^18^F-FDG PET or PET/CT

**Figure 1:**
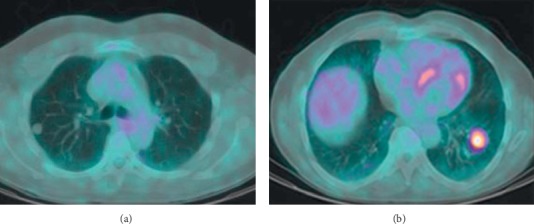
Two examples of solitary pulmonary nodules characterized by ^18^F-FDG PET/CT. (a) Axial ^18^F-FDG PET/CT image showing absence of significant radiopharmaceutical uptake in a pulmonary nodule of the right lung with a diameter of about 1 cm. Histology showed a benign tumour. (b) Axial ^18^F-FDG PET/CT image showing increased radiopharmaceutical uptake in a pulmonary nodule of the left lung with a diameter of about 2 cm. Histology showed a malignant tumour.

Characterizing SPNs detected incidentally or, as is the case more recently, on CT screening for LC, is a major public health issue. In the last decade, a robust evidence has been produced on the use of single time point (STP) ^18^F-FDG PET or PET/CT in the characterization of SPNs [5–11]. Two examples of ^18^F-FDG PET/CT images in the characterization of SPNs are shown in Figure 1.

Deppen et al. [5] performed a meta-analysis to estimate the diagnostic accuracy of ^18^F-FDG PET or PET/CT for SPNs suspicious for LC. The pooled sensitivity and specificity of ^18^F-FDG PET or PET/CT were 89% and 75%, respectively, with significant heterogeneity across the studies. There was a 16% lower specificity in regions with endemic infectious lung disease (61%) compared with nonendemic regions (77%). In general, the sensitivity did not change appreciably by endemic infection status. Overall, the accuracy of ^18^F-FDG PET or PET/CT for diagnosing LC among SPNs was extremely heterogeneous. These data support the use of ^18^F-FDG PET to diagnose LC among SPNs in regions where pulmonary infections are not endemic.

Wang et al. [6] suggested in their analysis that ^18^F-FDG PET/CT providing high sensitivity (98.7%) and moderate specificity (58.2%) could be applied for early diagnosis of LC.

Recently, Ruilong et al. [7] carried out a meta-analysis on the performance of ^18^F-FDG PET/CT for the evaluation of SPNs reporting a pooled sensitivity and specificity of 82% and 81%, respectively. As significant heterogeneity was observed, and a subgroup analysis showed that the best results for sensitivity (90%) and accuracy (93%) were present in prospective studies. Overall, their analysis suggested that ^18^F-FDG PET/CT is a useful tool for detecting malignant pulmonary nodules qualitatively. Although current evidence showed moderate accuracy for ^18^F-FDG PET/CT in differentiating malignant from benign SPNs, further work needs to be carried out to improve its reliability.

Another meta-analysis on the same topic [8] reported a pooled sensitivity of 89% and a pooled specificity of 70% for the diagnosis of malignant SPNs by ^18^F-FDG PET/CT. The authors concluded that this method cannot replace the “gold standard” pathology by resection or percutaneous biopsy and larger studies are required for further evaluation.

Recently, Divisi et al. [9] confirmed these findings reporting a pooled sensitivity and specificity of 81.9% and 62.4%, respectively, suggesting that ^18^F-FDG PET/CT has good diagnostic accuracy in SPN evaluation, but it should not be considered as a discriminatory test rather than a method to be included in a clinical and diagnostic pathway.

Two recent meta-analyses compared the diagnostic performance of ^18^F-FDG PET/CT with other radiological methods. Jia et al. [10] performed an indirect comparison among ^18^F-FDG PET/CT and CT for differentiating benign and malignant SPNs. The pooled sensitivity and specificity for ^18^F-FDG PET/CT were 89% and 78%, respectively. The corresponding values for CT were 94% and 73%, respectively. No significant differences were observed between CT and ^18^F-FDG PET/CT about the diagnostic parameters; both imaging methods showed a moderate-to-high diagnostic value for differentiating benign and malignant SPNs.

Lastly, a recent meta-analysis investigated the diagnostic performance of ^18^F-FDG PET/CT compared with diffusion-weighted magnetic resonance imaging (DW-MRI) for distinguishing malignant and benign SPNs [11]. DW-MRI had a pooled sensitivity and specificity of 83% and 91%, respectively, compared with 78% and 81%, respectively, for ^18^F-FDG PET/CT. The authors concluded that the diagnostic performance of DW-MRI is comparable or superior to that of ^18^F-FDG PET/CT in the differentiation of malignant and benign pulmonary lesions.

### 3.3. Characterization of Solitary Pulmonary Nodules with Dual Time Point ^18^F-FDG PET or PET/CT

Several meta-analyses have also explored the potential use of a dual time point (DTP) ^18^F-FDG PET or PET/CT in differentiating malignant from benign SPNs, performing both standard and delayed PET scans [12–15].

Lin et al. [12] performed a meta-analysis to assess the potential value of DTP compared with STP ^18^F-FDG PET in differentiating malignant from benign SPNs. The authors found a significant heterogeneity among the studies and a statistically nonsignificant trend toward higher sensitivity with DTP ^18^F-FDG PET, at moderate levels of specificity, when compared with initial STP ^18^F-FDG PET. Although the results of this analysis do not support the routine use of DTP ^18^F-FDG PET in the differential diagnosis of SPNs, this technique may provide additional information in selected cases with equivocal results from initial scanning, but further prospective research is required to better define the potential benefits of DTP ^18^F-FDG PET.

On the same topic, Barger and Nandalur [13] reported that the pooled sensitivity of DTP ^18^F-FDG PET was 85% and the pooled specificity was 77%. Significant heterogeneity was found. DTP ^18^F-FDG PET demonstrated similar sensitivity and specificity to STP ^18^F-FDG PET in the diagnosis of SPNs. Therefore, the additive value of DTP ^18^F-FDG PET is questionable.

Zhang et al. [14] reported a pooled sensitivity and specificity of DTP ^18^F-FDG PET of 79% and 73%, respectively. The corresponding values for STP ^18^F-FDG PET were 77% and 59%, respectively. These findings confirmed the similar accuracy of DTP and STP ^18^F-FDG PET in the differential diagnosis of SPNs, even if DTP ^18^F-FDG PET appears to be more specific than STP ^18^F-FDG PET.

Lastly, a recent meta-analysis on the same topic [15] reported a pooled sensitivity of 80% and a pooled specificity of 75% for DTP ^18^F-FDG PET/CT in discriminating malignant and benign SPNs, similar to the diagnostic values of STP ^18^F-FDG PET/CT. The authors suggested that further high-quality research is required to explore the potential value of DTP ^18^F-FDG PET/CT in this setting.

### 3.4. ^18^F-FLT PET for Evaluation of Pulmonary Lesions

The potential role of PET with ^18^F-FLT, a biomarker of proliferation, in the evaluation of pulmonary lesions was assessed by two meta-analyses. Li et al. [16] compared the diagnostic performance of ^18^F-FLT PET with ^18^F-FDG PET in evaluating patients with pulmonary lesions. This meta-analysis showed that ^18^F-FLT PET had a higher specificity (70%), but lower sensitivity (81%) compared to ^18^F-FDG PET (sensitivity: 92%; specificity: 50%). Therefore, ^18^F-FLT and ^18^F-FDG together could add diagnostic confidence for pulmonary lesions.

Wang et al. [17] performed a meta-analysis on the same topic: the direct comparisons showed lower sensitivity (80% vs. 89%) yet higher specificity (82% vs. 66%) for ^18^F-FLT PET compared with ^18^F-FDG PET. Although ^18^F-FLT PET cannot replace ^18^F-FDG PET in detecting small and early LC, it may help to prevent patients with larger or inflammatory lesions from cancer misdiagnosis or even overtreatment.

### 3.5. ^18^F-FDG PET or PET/CT for Evaluation of Pleural Lesions

^18^F-FDG PET and PET/CT demonstrated to be accurate diagnostic imaging methods in the differential diagnosis between malignant and benign pleural lesions in patients with or without known cancer history; nevertheless, possible sources of false-negative and false-positive results should be kept in mind and it cannot replace histopathological evaluation [18–20]. In patients without known cancer, pooled sensitivity and specificity of ^18^F-FDG PET and PET/CT were 95% and 82%, respectively [18]. In patients with known cancer, pooled sensitivity and specificity of ^18^F-FDG PET and PET/CT were 86% and 80%, respectively [19]. Porcel et al. in their meta-analysis [20] demonstrated that semiquantitative ^18^F-FDG PET assessment had a significantly lower sensitivity for diagnosing malignant pleural effusions than visual assessments. The pooled sensitivity and specificity of ^18^F-FDG PET or PET/CT using qualitative interpretation were 91% and 64%, respectively; the same pooled estimates using semiquantitative interpretation for identifying malignant pleural effusions were 81% and 74%, respectively. The moderate accuracy of semiquantitative PET assessment precludes its routine recommendation for discriminating malignant from benign pleural effusions.

## 4. Discussion

Our overview demonstrates that there is increasing evidence about the diagnostic performance of PET or PET/CT with different radiotracers in patients with suspicious LC or PT with good diagnostic accuracy values for some indications (Table 1).

Overall, current evidence-based data demonstrated the following points:

- A good diagnostic performance of ^18^F-FDG PET or PET/CT as a selective screening modality for LC has been demonstrated; nevertheless, its role in this setting remains unknown.
- ^18^F-FDG PET or PET/CT has good diagnostic performance for the characterization of SPNs with higher sensitivity than specificity values. The use of ^18^F-FDG PET or PET/CT for the characterization of SPNs seems to be supported by evidence-based data in regions when the specificity of the method is not too low.
- There is a similar diagnostic performance among STP and DTP ^18^F-FDG PET or PET/CT for the characterization of SPNs. The routine use of DTP ^18^F-FDG PET or PET/CT for this indication is currently not supported by evidence-based data.
- Compared to ^18^F-FDG PET or PET/CT, ^18^F-FLT PET or PET/CT has a lower sensitivity and a higher specificity for characterization of pulmonary lesions. The routine use of ^18^F-FLT PET or PET/CT for this indication is currently not supported by evidence-based data.
- ^18^F-FDG PET or PET/CT has a good diagnostic performance for the characterization of pleural lesions with higher sensitivity than specificity values. Evidence-base data suggest a possible role of this imaging method in this setting, but it cannot replace histopathological examination.

Awareness of the results described in this evidence-based review may affect patient care by providing supportive evidence for more effective use of PET/CT with different radiotracers in patients with suspicious LC or PT. Nevertheless, we would like to point out that diagnostic performance of an imaging method is not a measure of clinical effectiveness and good diagnostic accuracy of PET or PET/CT with different radiotracers for a specific indication does not necessarily result in improved patient outcomes. According to health technology assessment (HTA) principles which are valid also for PET imaging [21], other factors beyond the diagnostic performance of a test should be taken into account to support the clinical usefulness of an imaging method as availability, safety, legal, organization and economic aspects, and cost-effectiveness. To this regard, a recent cost-effectiveness analysis by Lopci et al. demonstrated that, despite a higher average cost for outpatient's diagnostics, the implementation of ^18^F-FDG PET/CT in the workup of undetermined lung nodules results in reduced unnecessary harm and costs related to inpatient's procedures [22]. These findings are in line with those of previous cost-effectiveness analyses which reported that the additional information gained from ^18^F-FDG PET or PET/CT in the characterization of SPNs is worth the cost in the context of proper medical indications [23, 24]. Therefore, it is expected that ^18^F-FDG PET/CT is reported in the list of approved procedures for the investigation of SPNs in several countries [24]. On the other hand, it is expected that ^18^F-FDG PET/CT may not be cost-effective for early diagnosis of LC in regions or countries where the specificity of this method is low (i.e., in countries where infectious lung diseases are endemic) [25]. In summary, in countries with a low incidence of pulmonary inflammatory or infectious diseases and a high incidence of LC, the diagnostic workup of SPNs should include ^18^F-FDG PET/CT as a main pillar. In countries with a high incidence of pulmonary inflammatory or infectious diseases and a low incidence of LC, this diagnostic workup needs to be adapted [26].

No cost-effectiveness analyses are currently available for the use of ^18^F-FDG PET/CT as a screening method for LC, for the use of DTP ^18^F-FDG PET/CT or ^18^F-FLT PET/CT for characterization of SPNs, and for the use of ^18^F-FDG PET/CT for characterization of pleural lesions.

About international guidelines, the last version of National Comprehensive Cancer Network (NCCN) guidelines recommends the use of ^18^F-FDG PET/CT for characterization of solid SPNs with diameter >8 mm [27]; this indication was set taking into account the significant risk of false-negative findings of ^18^F-FDG PET/CT for small lesions and nonsolid nodules [27].

Some limitations of the included meta-analyses should be underlined because they could hamper the achievement of definitive conclusions on the diagnostic performance of PET or PET/CT with different radiotracers in patients with suspicious LC or PT. In some meta-analyses, there is a limited number of included original studies, and this could have influenced the statistical power of the meta-analysis. In several meta-analyses, a significant heterogeneity across studies was described; this potential bias could be due to differences among patients included, methods and reference standard used, quality, and study design among the different included articles [28]. Furthermore, publication bias was reported in some meta-analyses pointing out that studies with significant findings were more likely to be published than those reporting nonsignificant results [28].

As a suggestion for further research, large multicenter prospective studies and in particular more cost-effectiveness analyses comparing ^18^F-FDG PET/CT with other imaging modalities in patients with suspicious LC and PT are warranted.

## Figures and Tables

**Table 1:** Characteristics and main findings of included meta-analyses on the diagnostic performance of PET or PET/CT with different radiotracers for evaluation of patients with suspicious lung cancer or pleural tumours.

Topic	Authors	Year	Articles included	Patients (p) or lesions (l) included	Sensitivity (95% CI)	Specificity (95% CI)	LR+ (95% CI)	LR−(95% CI)	DOR (95% CI)
^18^F-FDG PET or PET/CT for lung cancer screening	Chien et al. [4]	2013	4	9199 (p)	83% (75–89)	91% (86–95)	NR	NR	NR

Characterization of SPNs by single time point ^18^F-FDG PET or PET/CT	Zhang et al. [14]	2013	8	415 (p)	77% (71.9–82.3)	59% (50.6–66.2)	1.97 (1.32–2.93)	0.37 (0.29–0.49)	6.39 (3.4–12)
				430 (l)					
	Deppen et al. [5]	2014	70	8511 (l)	89% (86–91)	75% (71–79)	NR	NR	NR
	Wang et al. [6]	2015	4	1330 (p)	98.7%	58.2%	NR	NR	NR
	Li et al. [16]	2015	7	301 (p)	92% (86–95)	50% (41–58)	2.01 (1.38–2.93)	0.17 (0.10–0.29)	10.72 (5.51–20.87)
	Wang et al. [17]	2016	10	351 (p)	89%	66%	NR	NR	NR
	Ruilong et al. [7]	2017	12	1297 (p)	82% (76–87)	81% (66–90)	4.3 (2.3–7.9)	0.22 (0.16–0.3)	17.6 (8.2–37.7)
				1301 (l)					
	Li et al. [8]	2018	20	1557 (p)	89% (87–91)	70% (66–73)	3.33 (2.35–4.71)	0.18 (0.13–0.25)	22.43 (12.5–40.1)
	Divisi et al. [9]	2018	12	1463 (p)	81.9% (79.4–84.3)	62.4% (58.2–66.5)	2.19 (1.95–2.44)	0.29 (0.25–0.33)	7.05 (5.5–8.9)
	Jia et al. [10]	2019	23	NR	89% (85–92)	78% (66–86)	3.97 (2.57–6.13)	0.15 (0.10–0.20)	24 (12.7–45.5)
	Basso Dias et al. [11]	2019	5	735 (p)	78% (70–84)	81% (72–88)	4.1 (2.6–6.5)	0.8 (0.19–0.40)	15 (7–32)

Characterization of SPNs by dual time point ^18^F-FDG PET or PET/CT	Lin et al. [12]	2012	11	778 (p)	NR	NR	NR	NR	NR
	Barger and Nandalur [13]	2012	10	816 (p)	85% (82–89)	77% (72–81)	2.7 (1.4–5.2)	0.26 (0.14–0.49)	11 (3.8–32.2)
				890 (l)					
	Zhang et al. [14]	2013	8	415 (p)	79% (74–84)	73% (65–79)	2.61 (1.96–3.47)	0.29 (0.21–0.41)	10.25 (5.8–18.1)
				430 (l)					
	Zhao et al. [15]	2016	13	962 (p)	80% (76–84)	75% (71–79)	2.57 (1.54–4.29)	0.28 (0.16–0.5)	10.01 (3.8–26.2)

Characterization of SPNs by ^18^F-FLT PET or PET/CT	Li et al. [16]	2015	7	301 (p)	81% (74–87)	70% (61–77)	4.01 (1.62–9.88)	0.27 (0.20–0.37)	12.58 (6.8–23.2)
	Wang et al. [17]	2015	17	548 (p)	80% (74–85%)	82% (74–88)	NR	NR	NR

Characterization of pleural lesions by ^18^F-FDG PET or PET/CT	Treglia et al. [18]	2014	11	NR	95% (92–97)	82% (76–88)	5.3 (2.4–11.8)	0.09 (0.05–0.14)	74 (34–161)
	Treglia et al. [19]	2014	8	360 (p)	86% (80–91)	80% (73–85)	3.7 (2.8–4.9)	0.18 (0.09–0.34)	27 (13–56)
	Porcel et al. [20]	2015	11	NR	91% (86–94)	67% (56–77)	2.83 (2.04–3.98)	0.14 (0.08–0.22)	22 (10.2–41.7)

^18^F-FDG = fluorine-18 fluorodeoxyglucose; ^18^F-FLT = fluorine-18 fluorothymidine; LR+ = positive likelihood ratio; LR− = negative likelihood ratio; DOR = diagnostic odds ratio; 95% CI = 95% confidence interval; NR = not reported; SPNs = solitary pulmonary nodules; PET = positron emission tomography; CT = computed tomography.
